# Association Between Lead Exposure and Red Blood Cell Folate Concentrations in U.S. Children Aged 2–17 Years: An Analysis of Data from NHANES 2007–2018

**DOI:** 10.3390/ijerph21121676

**Published:** 2024-12-17

**Authors:** Wenping Hu, Tanya Telfair LeBlanc, Perri Zeitz Ruckart, Quanza Shavonne Brooks-Griffin, Paul Allwood

**Affiliations:** National Center for Environmental Health, Centers for Disease Control and Prevention, Atlanta, GA 30341, USA

**Keywords:** red blood cell folate, blood lead level, children, NHANES

## Abstract

The objective of this study is to evaluate the impact of low blood lead levels (BLLs) on the red blood cell folate concentrations in U.S. children aged 2–17 years. All data were obtained from the National Health and Nutrition Examination Survey (NHANES) over six consecutive cycles from 2007–2008 to 2017–2018. A total of 12,739 children with BLLs lower than 10 µg/dL (geometric mean: 0.66 µg/dL) were included in the dataset. BLLs were categorized into three tertiles (tertile 1: <0.55 µg/dL; tertile 2: 0.55–0.95 µg/dL; and tertile 3: ≥0.95 µg/dL). The multivariate linear regression model analysis indicates a negative relationship between BLLs and red blood cell folate concentrations. After adjusting for potential confounding factors, red blood cell folate concentrations were lower in children in the BLL tertile 2 (β-coefficient = −0.0450; 95% CI: −0.0676, −0.0224) and BLL tertile 3 groups (β-coefficient = −0.0775; 95% CI: −0.1032, −0.0517) compared to children in the BLL tertile 1 group. When stratified by age, gender, and race/Hispanic origin, the subgroup analysis consistently revealed a negative relationship between BLLs and red blood cell folate concentrations, with red blood cell folate concentrations being lower (*p* < 0.05) in children in the BLL tertile 3 group compared to children in the tertile 1 group. Further investigation is needed to explore the mechanism underlying the potential relationship between BLLs and red blood cell folate concentrations and determine whether folate plays an active role beneficial for preventing the harmful effects of lead on children.

## 1. Introduction

Lead is a toxic heavy metal occurring in the environment, and its various industrial and domestic usages pose an enormous risk to public health [1]. Exposure to lead adversely affects the human body systems. Children, particularly those younger than 6 years of age, are at high risk of lead exposure and poisoning [2,3]. Over the past several decades, the United States have seen a dramatic and sustained decline in population-level lead exposure because of lead source control measures, such as the removal of lead from gasoline and paint [4]. Nevertheless, lead cannot be eliminated from the environment, and lead sources remain plentiful; thus, many U.S. children still experience low-level exposure to lead [3]. Meanwhile, evidence continues to indicate the adverse effects of lead at progressively decreasing amounts of exposure [5].

Folate is a micronutrient that occurs naturally in many foods but that is unable to be synthesized by the human body. It plays an essential role in body cell growth and development, involving many metabolic pathways [6]. For instance, folate is a methyl group donor for the synthesis of methionine from homocysteine catalyzed by the vitamin B12-dependent methionine synthetase. The health effects of folate are well recognized. For example, the risk of neural tube defects increases with folate insufficiency. Metabolic changes due to an impaired folate status have been associated with an increased risk of chronic disease development, including cancer, cardiovascular diseases, and cognitive dysfunction [7].

Several studies have been conducted to examine the potential relationship between serum folate concentrations and blood lead levels (BLLs) [8,9] and between BLLs and serum/red blood cell folate concentrations [10,11]; from those studies, it appears that lead exposure is negatively associated with folate status assessed by serum/red blood cell folate concentration. Nevertheless, such an association might differ depending on the different age groups evaluated, the lead exposure levels, and the analytical method used for assessing the folate status. The research on the association between low BLLs and red blood cell folate concentrations in children is limited. Therefore, the objective of the present study is to explore the potential relationship between low BLLs and folate status in U.S. children aged 2–17 years, using the National Health and Nutrition Examination Survey (NHANES) data from 2007 to 2018.

## 2. Methods

### 2.1. Study Population

The National Health and Nutrition Examination Survey is conducted by the National Center for Health Statistics, Centers for Disease Control and Prevention (CDC). Details regarding the survey and data collection procedures are available on the NHANES website [12]. The data from six consecutive cycles of NHANES (i.e., 2007–2008, 2009–2010, 2011–2012, 2013–2014, 2015–2016, and 2017–2018) were combined. A total of 12,753 children aged 2–17 years without missing values of sample weight, BLLs, and red blood cell folate concentrations were selected, of which 14 children who had BLLs ≥ 10 µg/dL were excluded. Ultimately, a total of 12,739 children were included in the analysis.

### 2.2. Measurement of Blood Lead

Whole blood specimens were obtained and transferred into blood collection tubes by venipuncture from eligible children during the health examination, maintained at refrigerated temperatures (2–8 °C), transferred to plastic, pre-screened cryovials before freezing, and stored frozen at ≤−70 °C until analysis. All sample collection, storage, and analysis consumables were pre-screened prior to use to ensure that they were free of significant lead contamination. Blood lead content was measured via inductively coupled plasma mass spectrometry [13]. The limit of detection for blood lead decreased from 0.25 µg/dL in 2007–2012 to 0.07 µg/dL in 2013–2018 as laboratory technology improved. NHANES has identified the results below the limit of detection and replaced them with a value equal to the detection limit divided by the square root of 2 [12].

### 2.3. Measurement of Red Blood Cell Folate

Whole blood folate was measured through a microbiological assay, while serum folate was measured either through a microbiological assay (2007–2010) or through isotope dilution high-performance liquid chromatography coupled with tandem mass spectrometry (LC-MS/MS) (2011–2018) [12]. Red blood cell folate was calculated using the data from both assays. In the microbiological assay, diluted whole blood or serum was added to a medium inoculated with *Lactobacillus rhamnosus*, containing all nutrients necessary for the growth of *Lactobacillus rhamnosus* except for folate. After incubating the inoculated medium for 45 h at 37 °C, the folate concentration was determined by measuring the turbidity of the inoculated medium at 590 nm using a PowerWave X340 microplate reader (Bio-Tek Instruments Inc., Winooski, VT, USA). The assay was calibrated with 5-methyl-tetrahydrofolate from Merck & Cie (Schaffhausen, Switzerland). In the assay for serum folate by LC-MS/MS, five folate forms (i.e., 5-methyl-tetrahydrofolate, folic acid, 5-formyl-tetrahydrofolate, tetrahydrofolate, and 5,10-methenyl-tetrahydrofolate) were measured. Serum folate was then calculated as the sum of the five individual folate forms [12]. The sum of the folate forms determined by LC-MS/MS corresponded well with the total folate determined through the microbiological assay, making these two assays interchangeable [14,15].

### 2.4. Other Variables

Variables such as age, gender (male vs. female), race/Hispanic origin, ratio of family income to poverty (PIR), health insurance (yes vs. no), education level of the household reference person, second-hand smoke (≥1 smoker vs. no smoker in the household), and body mass index (BMI) were included in the regression analysis. The age of the children was categorized as 2–5 years, 6–11 years, and 12–17 years. The race/Hispanic origin was categorized as Hispanic, non-Hispanic white, non-Hispanic black, and other. PIR, the ratio of family income to poverty threshold as defined by the U.S. Census Bureau, was categorized as <1, 1–2, and ≥2. The education level of the household reference person was categorized as follows: less than high school, high school or some college, and college graduate or above. BMI is a measure of body weight (in kilograms) divided by the square of the height (in meters). Based on sex-specific BMI-for-age CDC growth charts [16], children with BMI values lower than the 5th percentile, between the 5th and the 85th percentile (excluded), between the 85th and the 95th percentile (excluded), and the 95th percentile of the growth charts were categorized as underweight, normal weight, overweight, and obesity, respectively. For variables (PIR, health insurance, education level of the household reference person, second-hand smoke, and BMI) with unknown values, all unknown values were considered as one category in its respective variable and included in the regression analysis.

### 2.5. Statistical Analyses

We conducted multivariate linear regression analyses to evaluate the potential relationships between BLLs (predictor) and red blood cell folate concentrations (outcome). There were two models in the present study: (I) a crude model, not adjusted for covariates, and (II) an adjusted model, adjusted for age, gender, race/Hispanic origin, second-hand smoke, PIR, health insurance, education level of the household reference person, and BMI. Red blood cell folate concentrations and the age of the children were continuous variables in the overall model and subgroup model analyses. BLLs were categorized into three tertiles (i.e., tertile 1: <0.55 µg/dL; tertile 2: 0.55–0.95 µg/dL; and tertile 3: ≥0.95 µg/dL). BLLs and red blood cell folate concentrations were not normally distributed; thus, log-transformed values of BLLs and red blood cell folate concentrations were used in the multivariate linear regression analysis. All statistical analyses were conducted using the Survey Sampling and Analysis Procedures in SAS (version 9.4, SAS Institute Inc., Cary, NC, USA) to account for the complex design and sample weight from NHANES. The sample weights across multiple survey cycles were constructed based on the sample weights from the selected survey cycles of NHANES and, specifically, folate and folate forms weight from the survey cycle 2017–2018. The subgroup analyses were performed using a stratified multivariate regression model by specifying each subgroup (i.e., age, gender, race/Hispanic origin) in the DOMAIN statement. Significance was declared at a *p*-value of <0.05.

## 3. Results

The BLLs and red blood cell folate concentrations according to the characteristics of the study population are shown in Table 1. There were 51.1% males and 48.9% females, 18.6% children aged 2–5 years, 41.6% children aged 6–11 years, and 39.7% children aged 12–17 years. The race/Hispanic origin of the children was categorized as Hispanic (24.0%), non-Hispanic white (52.5%), non-Hispanic black (14.1%), and other (9.4%) (Table 1). The overall geometric mean (GM) of BLLs and red blood cell folate concentrations was 0.66 µg/dL and 462 ng/mL, respectively. It appeared that the GM of the red blood cell folate concentrations decreased with increasing BLLs, from 473 ng/mL in the BLL tertile 1 (<0.55 µg/dL; GM: 0.37 µg/dL) to 459 ng/mL in the BLL tertile 2 (0.55–0.95 µg/dL; GM: 0.71 µg/dL), further decreasing to 451 ng/mL in the BLL tertile 3 (≥0.95 µg/dL; GM: 1.50 µg/dL) (Table 1).

**Figure 1:**
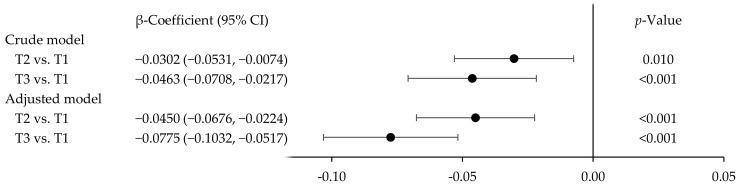
Regression β-coefficients [95% confidence interval (CI)] for the association between blood lead levels (T1: tertile 1, <0.55 µg/dL; T2: tertile 2, 0.55–0.95 µg/dL; T3: tertile 3, ≥0.95 µg/dL) and red blood cell folate concentrations in children aged 2–17 years. Data from NHANES 2007–2018.

The potential relationship between BLLs and red blood cell folate concentrations is presented in Figure 1. For the crude model, the β-coefficients for the children in the BLL tertiles 2 and 3 were −0.0302 [95% confidence interval (CI): −0.0531, −0.0074; *p* = 0.010] and −0.0463 (95% CI: −0.0708, −0.0217; *p* < 0.001), respectively, compared to the children in the BLL tertile 1. In the adjusted model, the β-coefficients for the children in the BLL tertiles 2 and 3 were −0.0450 (95% CI: −0.0676, −0.0224; *p* < 0.001) and −0.0775 (95% CI: −0.1032, −0.0517; *p* < 0.001), respectively. Both the crude and the adjusted models showed a negative association between BLLs and red blood cell folate concentrations (Figure 1).

We further explored the potential relationship between BLLs and red blood cell folate concentrations based on age, gender, and race/Hispanic origin via subgroup analysis (Table 2). In the adjusted model, the negative association between BLLs and red blood cell folate concentrations was statistically significant (*p* < 0.05) in the children within the BLL tertile 3 compared to those in the tertile 1, regardless of the different groups of age, gender, and race/Hispanic origin. With respect to the children in the BLL tertile 2 compared to those in the tertile 1, such a negative association was still statistically significant (*p* < 0.05), except for those in the following groups: age of 2–5 years, non-Hispanic black, and the race/Hispanic origin group “other” (Table 2).

## 4. Discussion

Lead is toxic to the human body; children are particularly vulnerable to lead poisoning, as they are more likely to be exposed to various lead sources and their absorption from ingested lead is higher than that in adults [17]. Exposure to lead, even at low levels, induces physical, intellectual, and/or behavioral human health problems [18,19,20]. For instance, Evens et al. [19] found that BLLs below 10 µg/dL were inversely associated with reading and math scores in children enrolled in the third grade of Chicago public schools, and estimated that 13% of reading failures and 14.8% of math failures could be attributed to lead exposure resulting in BLLs of 5–9 µg/dL vs. 0–4 µg/dL. In the present study, we focused on exploring the potential relationship between BLLs, specifically at low levels (range: 0.05–<10 µg/dL; GM: 0.66 µg/dL), and folate status. Our results indicate that exposure to lead at low levels is, indeed, inversely associated with red blood cell folate concentrations in U.S. children.

Several studies have investigated the folate status in adults suffering from lead exposure [9,10,11]. Consistent with our observations, Wang et al. [11] used NHANES data (2017–2018, *n* = 5690) and found that BLLs were negatively associated with red blood cell folate concentrations among adults, and such an association was stronger when the highest lead tertile group was compared with the lowest lead tertile group. Büyükşekerci et al. [10] conducted a study on 944 patients whose BLLs and serum folate concentrations were assessed, with the patients classified into a lead-exposed group with BLLs over 10 µg/dL (median: 34.15 µg/dL; range: 10.3–116 µg/dL) and a control group with BLLs below 10 µg/dL (median: 2.3 µg/dL; range: 0.01–9.82 µg/dL). The median serum folate concentration was significantly lower in the lead-exposed group compared to the control group (6.3 ng/mL vs. 6.8 ng/mL; *p* < 0.001). Outside of the adult population, He et al. [8] explored the potential relationship between serum folate concentrations and BLLs in adolescents aged 12–19 years. Similarly, they reported a negative relationship between serum folate concentrations and BLLs. It should be noted that the NHANES data used in their study [8] included mostly children aged 12–17 years that were also included in the present study; moreover, serum, rather than red blood cell, folate concentration was used to evaluate the folate status. Serum folate is a sensitive indicator of recent folate intake from the diet, and repeated measurements may be needed over time to assess the long-term status. In contrast, red blood cell folate responds slowly to changes in dietary folate intake, and, thus, it is indicative of a long-term folate status [21,22]. Although a moderate-to-strong correlation has been found between serum and red blood cell folate, serum and red blood cell folate concentrations may provide different information on the folate status [23]. Nevertheless, it appears that the BLL is inversely associated with the folate status in children, regardless of whether the folate status is assessed by measuring the serum or the red blood cell folate concentration.

No mechanistic explanation is readily apparent for the inverse association between BLLs and red blood cell folate concentrations. Oxidative stress induced by lead exposure might be one of the major mechanisms underlying lead toxicity. Oxidative stress occurs when the production of reactive oxygen species exceeds the capacity of the antioxidants against those free radicals. It has been demonstrated that antioxidants such as glutathione play an essential role in protecting the cells through the direct interaction of their sulfhydryl group with the free radicals; therefore, the intracellular glutathione becomes depleted during the process against the lead-induced oxidative stress [24,25]. On the contrary, micronutrient folate mediates the one-carbon metabolism, a central metabolic hub, providing one-carbon units for the essential synthesis of DNA and amino acids such as methionine [26]. Methionine may, subsequently, be used for glutathione synthesis through the transsulfuration pathway. There exists evidence that folate serves as a redox regulator by upregulating reactive oxygen species sinker proteins to combat reactive oxygen species insults and averts mitochondrial glutathione depletion by positively regulating glutathione biosynthesis, the glutathione transporting system, and the mitochondrial glutathione recycling process [27]. In addition, Joshi et al. [28] reported that folic acid (the synthetic form of folate) effectively scavenges free radicals and inhibits lipid peroxidation.

The present study is subject to several limitations. First, the cross-sectional analysis of NHANES data permitted no inferences of causality. In our view, randomized controlled animal trials should be conducted to prove causality, that is, that red blood cell folate concentrations are indeed impacted by BLLs. Second, we could not rule out unincluded and unmeasured confounding factors as an alternative explanation for the findings. Third, missing values occurred for BLLs and red blood cell folate concentrations, with the missing data possibly causing a selection bias. Fourth, NHANES has been designed as a nationally representative sample of the U.S. civilian, non-institutionalized population. The findings from the present study are generalizable to children within the United States only, but not worldwide. Mandatory folic acid fortification has been implemented in the United States since 1998 [29]. However, Quinn et al. [30] reported that, among 193 countries examined up to 2023, 77 countries had no folate fortification program, accounting for 15% of the worldwide population, and that folic acid fortification, worldwide, was associated with 50–100% higher population mean plasma folate levels and a 25–50% lower neural tube defects prevalence compared to the population subject to no fortification. The inverse association between BLLs and red blood cell folate concentrations demonstrated in the present study implies that an interaction between lead exposure and micronutrient folate in children possibly occurs. It could be anticipated that the risk of folate deficiency associated with lead exposure in children would increase in countries without effective folate fortification policies.

## 5. Conclusions

In conclusion, higher BLLs are associated with lower red blood cell folate concentrations in U.S. children aged 2–17 years, based on a cross-sectional analysis of NHANES data. Further research is needed to investigate the mechanism underlying the interaction of BLL with red blood cell folate.

## Figures and Tables

**Table 1:** Blood lead levels and red blood cell folate concentrations in children aged 2–17 years (*n* = 12,739) stratified by study characteristics. Data from NHANES 2007–2018.

Variable	*n*	% (Weighted) ^a^	Lead GM (SE) ^b^, µg/dL	Folate GM (SE) ^b^, ng/mL
Gender				
Male	6477	51.1	0.72 (0.01)	466 (4)
Female	6262	48.9	0.60 (0.01)	459 (3)
Age				
2–5 years	3111	18.6	0.96 (0.02)	482 (5)
6–11 years	5563	41.6	0.66 (0.01)	490 (3)
12–17 years	4065	39.7	0.56 (0.01)	427 (5)
Race/Hispanic origin				
Hispanic	4594	24.0	0.63 (0.02)	457 (3)
Non-Hispanic white	3500	52.5	0.64 (0.02)	482 (5)
Non-Hispanic black	3092	14.1	0.80 (0.02)	406 (4)
Other	1553	9.4	0.68 (0.02)	462 (8)
Ratio of family income to poverty				
<1	4084	23.2	0.81 (0.01)	453 (5)
1–2	3370	23.4	0.70 (0.02)	462 (5)
≥2	4353	47.4	0.58 (0.01)	469 (4)
Unknown	932	6.0	0.67 (0.03)	443 (8)
Health insurance				
Yes	11,534	92.0	0.65 (0.01)	463 (3)
No	1179	7.9	0.74 (0.02)	452 (8)
Unknown	26	0.1	0.84 (0.15)	394 (31)
Education ^c^				
Less than high school	3472	20.1	0.75 (0.02)	448 (5)
High school or some college	6456	50.8	0.69 (0.01)	458 (4)
College graduate or above	2397	25.8	0.55 (0.01)	484 (5)
Unknown	414	3.2	0.64 (0.04)	461 (10)
Second-hand smoke				
≥1 smoker in household	2460	19.6	0.74 (0.02)	454 (6)
No smoker in the household	10,157	79.4	0.64 (0.01)	465 (3)
Unknown	122	1.0	0.65 (0.06)	446 (33)
Body mass index				
Underweight	377	3.3	0.70 (0.05)	483 (17)
Normal weight	7723	61.6	0.68 (0.01)	462 (4)
Overweight	2058	16.2	0.62 (0.01)	460 (5)
Obesity	2449	18.1	0.61 (0.01)	463(5)
Unknown	132	0.8	0.85 (0.06)	461 (19)
Blood lead level (µg/dL)				
Tertile 1 (<0.55)	4190	41.2	0.37 (0.00)	473 (5)
Tertile 2 (0.55–0.95)	4339	33.0	0.71 (0.00)	459 (4)
Tertile 3 (≥0.95)	4210	25.9	1.50 (0.02)	451 (4)
Total	12,739	100.0	0.66 (0.01)	462 (3)

^a^ The totals may not precisely add up due to rounding. ^b^ GM (SE): geometric mean (standard error). ^c^ Education: education level of the household reference person.

**Table 2:** Regression β-coefficients (95% CI) for red blood cell folate concentrations by tertile of blood lead levels, stratified by age, gender, and race/Hispanic origin in children aged 2–17 years. Data from NHANES 2007–2018 ^a b c^.

		**Crude Model**		**Adjusted Model** ^a^	
		**β-Coefficient (95% CI)** ^c^	***p*-Value**	**β-Coefficient (95% CI)** ^c^	***p*-Value**
Age					
2–5 years	T2 vs. T1 ^b^	−0.0484 (−0.0819, −0.0149)	0.005	−0.0260 (−0.0598, 0.0079)	0.131
	T3 vs. T1 ^b^	−0.1213 (−0.1529, −0.0897)	<0.001	−0.0791 (−0.1118, −0.0463)	<0.001
6–11 years	T2 vs. T1	−0.0534 (−0.0776, −0.0292)	<0.001	−0.0490 (−0.0724, −0.0255)	<0.001
	T3 vs. T1	−0.0630 (−0.0908, −0.0351)	<0.001	−0.0488 (−0.0762, −0.0214)	<0.001
12–17 years	T2 vs. T1	−0.0467 (−0.0873, −0.0060)	0.025	−0.0474 (−0.0885, −0.0063)	0.024
	T3 vs. T1	−0.0774 (−0.1176, −0.0372)	<0.001	−0.0802 (−0.1216, −0.0389)	<0.001
Gender					
Male	T2 vs. T1	−0.0400 (−0.0754, −0.0046)	0.027	−0.0466 (−0.0798, −0.0134)	0.006
	T3 vs. T1	−0.0503 (−0.0822, −0.0184)	0.002	−0.0627 (−0.0949, −0.0304)	<0.001
Female	T2 vs. T1	−0.0249 (−0.0487, −0.0011)	0.041	−0.0436 (−0.0675, −0.0197)	<0.001
	T3 vs. T1	−0.0513 (−0.0826, −0.0200)	0.002	−0.0999 (−0.1310, −0.0687)	<0.001
Race/Hispanic origin					
Hispanic	T2 vs. T1	−0.0240 (−0.0494, 0.0013)	0.063	−0.0403 (−0.0653, −0.0153)	0.002
	T3 vs. T1	−0.0216 (−0.0506, 0.0074)	0.142	−0.0470 (−0.0791, −0.0150)	0.005
Non-Hispanic white	T2 vs. T1	−0.0239 (−0.0622, 0.0144)	0.218	−0.0468 (−0.0852, −0.0083)	0.018
	T3 vs. T1	−0.0265 (−0.0682, 0.0151)	0.209	−0.0790 (−0.1238, −0.0342)	<0.001
Non-Hispanic black	T2 vs. T1	0.0054 (−0.0333, 0.0442)	0.781	−0.0287 (−0.0681, 0.0107)	0.152
	T3 vs. T1	0.0140 (−0.0251, 0.0531)	0.480	−0.0616 (−0.1052, −0.0181)	0.006
Other	T2 vs. T1	−0.0418 (−0.1067, 0.0231)	0.204	−0.0616 (−0.1232, 0.0000)	0.050
	T3 vs. T1	−0.1323 (−0.2007, −0.0639)	<0.001	−0.1465 (−0.2071, −0.0860)	<0.001

^a^ Adjusted model: adjusted for age (continuous), gender, race/Hispanic origin, second-hand smoke, ratio of family income to poverty, health insurance, education level of the household reference person, and body mass index. ^b^ Three tertiles (T1–T3) of blood lead levels: T1, <0.55 µg/dL; T2, 0.55–0.95 µg/dL; T3, ≥0.95 µg/dL. ^c^ 95% CI: 95% confidence interval.

## Data Availability

All data are presented in the article. For further information, contact the corresponding author.
